# Plant RNA Binding Proteins as Critical Modulators in Drought, High Salinity, Heat, and Cold Stress Responses: An Updated Overview

**DOI:** 10.3390/ijms22136731

**Published:** 2021-06-23

**Authors:** Muthusamy Muthusamy, Jong-Hee Kim, Jin A Kim, Soo-In Lee

**Affiliations:** 1 Department of Agricultural Biotechnology, National Institute of Agricultural Sciences (NAS), RDA, Jeonju 54874, Korea; biotech.muthu@gmail.com (M.M.); gllmon@naver.com (J.-H.K.); jakim72@korea.kr (J.A.K.); 2 Division of Horticultural Biotechnology, Hankyung National University, Anseong 17579, Korea

**Keywords:** plant RNA binding proteins, abiotic stress, RNA metabolism, post transcriptional gene regulation

## Abstract

Plant abiotic stress responses are tightly regulated by different players at multiple levels. At transcriptional or post-transcriptional levels, several RNA binding proteins (RBPs) regulate stress response genes through RNA metabolism. They are increasingly recognized as critical modulators of a myriad of biological processes, including stress responses. Plant RBPs are heterogeneous with one or more conservative RNA motifs that constitute canonical/novel RNA binding domains (RBDs), which can bind to target RNAs to determine their regulation as per the plant requirements at given environmental conditions. Given its biological significance and possible consideration as a potential tool in genetic manipulation programs to improve key agronomic traits amidst frequent episodes of climate anomalies, studies concerning the identification and functional characterization of RBP candidate genes are steadily mounting. This paper presents a comprehensive overview of canonical and novel RBPs and their functions in major abiotic stresses including drought, heat, salt, and cold stress conditions. To some extent, we also briefly describe the basic motif structure of RBPs that would be useful in forthcoming studies. Additionally, we also collected RBP genes that were modulated by stress, but that lacked functional characterization, providing an impetus to conduct further research.

## 1. Introduction

Plant RNA-binding proteins (RBPs) are heterogeneous, ubiquitous in nature, and control gene regulation, both co- and post-transcriptionally, by coordinating the regulation of RNA metabolism in response to perceived signals from within and in the environment. Therefore, RBPs are considered one of the several layers of crucial determinants of gene regulation in eukaryotic plants. Plants encode over 1800 RBPs known to modulate a myriad of steps in gene regulation, from chromatin organization to translation [1]. The gene regulation of RBPs is specified by their diverse RNA-binding domains (RBDs) and by their interaction with pre-mRNA targets. Based on the regulatory or biological functions of target RNAs, the functions of RBD-containing genes were specified. The direct or indirect interactive nature of the RBPs with single/double-strand RNA molecules has a critical role in RNA biogenesis (primarily as RNA chaperones) [2] and the overall functioning of plants/cells, which helps in the adaptation of plants to various developmental and environmental conditions [3]. The diverse RBPs found in plants have characteristic well-conserved one or more RBDs (canonical/novel) at the N-terminus and various auxiliary motifs at the c-terminal end [4]. Each RBD and its combined interaction with the untranslated/cis-acting regulatory regions of target mRNAs decides the functional specificity of RBPs according to plant requirements [5].

The application of high-throughput approaches like mRNA-protein interactome capture [6,7], other large-scale proteome analyses and bioinformatics-based annotation pipelines [3,8] has immensely accelerated the identification of RBPs, which proportionally increased the RBPs profile in model and non-model plants. As a result, several canonical RBDs, including RNA Recognition Motif (RRM), hnRNP K Homology (KH) domain, zinc finger motif (CCCH type), YT512-B Homology (YTH) domain, cold-shock domain (CSD), pentatricopeptide repeat (PPR), DEAD/DEAH RNA helicases, S1 RNA-binding domain, chloroplast ribosome maturation and splicing domain (CRM), double-stranded RNA-binding domain (DS-RBD), Pumilio/fem-3 binding factors (PUF) RNA-binding domain, Piwi/Argonaute/Zwiklle (PAZ) domain, nuclear transport factor 2, and like-Smith (commonly known as LSm) poly (A) binding domains were identified in plants [3,6,9,10,11,12,13,14,15,16,17,18,19,20,21,22,23]. These canonical RBD domains containing proteins can be categorized into several subclasses based on the auxiliary domains present at the c-terminals. Some of these motifs are rich in particular amino acids like glycine (hence known as glycine-rich), serine–arginine (SR)/arginine–serine (RS) repeats, arginine (arginine-rich), or zinc finger motif [24]. Additionally, recent merging studies show that several other RBPs with non-canonical RBDs were continuously being identified. Heterogeneous nuclear RNA-binding protein-like [25], Nuclear Speckle RNA Binding Proteins [26], zipcode RNA binding [27], ABA-regulated RNA-binding protein (ARP1) [28], cap-binding complex (CBC) [29], and zinc finger proteins like Zf-C2H2, Zf-CCHC, Zf-RING, Zf-PHD, and Zf-RanBP [3] are some of the non-canonical RBPs found in plants. In Arabidopsis, the most represented non-canonical RBDs are the ribosomal proteins playing extra-ribosomal functions in mRNA regulation [3]. The emergence of new RBP classes is ultimately revealing the complexity of RBP mediated gene regulation in plants [17]. Most of the identified RBPs function as RNA chaperones that govern many aspects of RNA metabolism, including pre-mRNA processing/editing, transport, stability/decay, and translation [3,20,21,30,31,32]. Having a role in alternative splicing, RBPs are likely to modulate more than 60% of the plant transcriptome [33,34,35] that regulates plant growth, development, and stress responses, which indicates the importance of RBPs. Therefore, RBPs are increasingly recognized as post-transcriptional regulators [5] and are being utilized for improving key agronomic traits, including yield, biomass, and stress tolerance.

## 2. Regulation of RBPs

**Figure 1:**
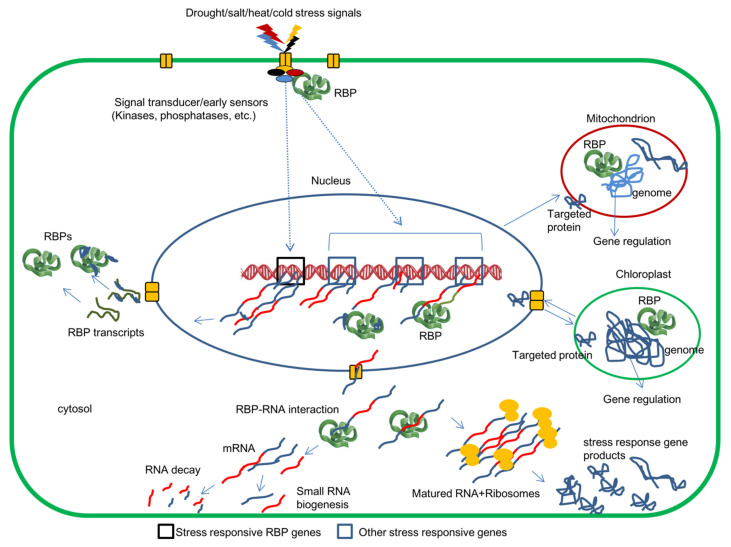
A simplified diagrammatic representation of activation of RBP and other genes by abiotic stress signals (drought, salt, heat, and cold) and RBP interactions with RNAs of nuclear, chloroplast, and the mitochondrion at various levels to regulate stress responses in a plant cell. The stress signals perceived by the sensors were transduced to the nucleus, where activation of several genes, including RBP genes, was happening to adjust the cellular conditions to stress. The RBPs interact with various stress response transcripts and modulate their expression by altering their stability, splicing, cellular localization, availability/degradation, and translational repressions.

The expression of RBPs and their association with target mRNAs is dynamic and subject to changes according to signals/stresses perceived by the plant cells. Stresses on plants can elicit RBP dependent stabilization of key mRNAs whose functions are essential for maintaining cellular activities and alleviating the stress damages caused to the plant cell. The stress-induced RBPs can also destabilize mRNAs that are unwanted under stressful conditions. Although the precise mechanism responsible for stress-responsive RBP activation is limited, multiple lines of available evidence suggest that stress-responsive alternative splicing at post-transcriptional levels decides the activation/deactivation of RBPs under adverse conditions [34,36,37]. The early stress-response regulators are expected to perceive the primary stress signals and transduce them to the nucleus, where the upstream abiotic stress response cis-elements of RBP genes were targeted for its regulation [20,36,38] (Figure 1). The activity of existing RBPs can be transiently regulated by post-translational modifications and, indeed, RBPs are enriched in post-translational modification (PTM) sites [5,7]. The different RBPs and their role in drought, high salinity, heat/high-temperature, and cold/low-temperature stress conditions are listed in Table 1, Table 2, Table 3 and Table 4, respectively.

**Table 1:** Plant RNA binding proteins implicated in drought stress responses.

RBPs	Study	Total Genes	Species	Drought	Modulation		Ref.
					Upregulated	Downregulated	
YTH	*-*	39	*T. aestivum* L	-	responsive		[39]
	*-*	10	*C. sinensis*	-	*CitYTH2, 3, 5, 7, 8*	*CitYTH1, CitYTH4*	[40]
	*MhYTP1*	26	*M. domestica*	T	*-*	*-*	[41]
	*-*	26	*M. domestica*	-	responsive		[42]
	*-*	13	*A. thaliana*	-	*-*	*AtYTH10*	[18]
	*-*	12	*O. sativa*	-	*OsYTH01, 2, 3,5,12*	*OsYTH10, 11*	[18]
SR	*-*	18	*M. esculenta* Crantz	-	*MeRS40*	*MeSR20, Z21a, 34a, RSZ22a, SC35*	[43]
	*-*	18	*B. distachyon*	-	responsive		[44]
	*SR45a*		*B. rapa*	T	*SR45a*	*-*	[45]
GR-RBP	*GRP7*	8	*A. thaliana*	S	*-*	*-*	[46]
	*-*	37-Ga 32-Gr	*G. arboretum* *G. raimondii*	-	*GaRB-GRP4, 9,17*	*-*	[47]
	*OsGRP3*	-	*O. sativa*	T	*OsGRP3*	*-*	[48]
	*BrRZ1, 2, 3*	-	*B. rapa*	S	*BrRZ1, BrRZ2, BrRZ3*	*-*	[21]
	*TaRZ2 or 3*	-	*T. aestivum*	S	*-*	*-*	[49]
	*CSDP1*	-	*A. thaliana*	S	*-*	*-*	[50]
	*CspA*	-	*E. coli*/ *A. thaliana*	T	*-*	*-*	[51]
PPR	*SOAR1*	-	*A. thaliana*	T	*-*	*-*	[52]
	*-*	491	*O. sativa*	-	*LOC_Os02g46980,* *LOC_Os04g01990,* *LOC_Os03g53170*	*-*	[53]
	*GmPPR4*	179 DYW	*G. max*	T	*GmPPR4*	*-*	[54]
	*POCO1*	-	*A. thaliana*	S (M)	*-*	*-*	[55]
DEAD-RH	*SlDEAD31*	-	*S. lycopersicum*	T	*SlDEAD31*	*-*	[56]
	*OsRH58*	-	*O. sativa*	T	*OsRH58*	*-*	[32]
	*OsABP*		*O. sativa*	-	*OsABP*		[57]
	*ARP1*	-	*A. thaliana*	S	*-*	*-*	[28]
PUF	*APUM5*		*A. thaliana*	S			[58]
	*hnRNP-like*	-	*E. guineensis*	-	*EgRBP42*	*-*	[25]

Note: T, stress tolerance; S, stress sensitive/susceptible; T (M), stress-tolerant mutant; S (M), stress-sensitive mutants; “-”, not available.

**Table 2:** Plant RNA binding proteins implicated in high-salinity stress responses.

RBP (Domain)	Study	Total Genes	Species	Salinity	Modulation		Ref.
					UP	Down	
YTH	***-***	39	*T. aestivum* L	-	responsive		[39]
	*-*	10	*C. sinensis*	-	*CitYTH4*	*CitYTH2*	[40]
	*-*	5	*C. sativus*	-	*-*	*CsYTH1, 2, 3, 4, 5*	[38]
	*-*	26	*M. domestica*	-	responsive		[42]
	*-*	13	*A. thaliana*	-	*-*	*AtYTH10*	[18]
SR	*SR45a*		*A. thaliana*	S	*SR45a-1a, SR45a-1b*	*-*	[34]
	*SR45.1*		*A. thaliana*	T			[59]
	*MeSR34*	18	*M. esculenta* Crantz	T	*MeRS40,* *MeRS31,* *MeRS2Z33*	*MeRSZ21a, MeSCL28, MeRS2Z33, MeSR34a, MeRSZ22a, MeSC35*	[43]
	*-*	18	*B. distachyon*	-	responsive		[44]
	*-*	28	*B. rapa*	-	*BrSR3,* *BrSCL2,* *BrSR-like 3*	*-*	[20]
GR-RBP	*NtRGP-1a, 1b, 2, 3*		*N. tabacum*	-	*-*	*NtRGP-1a, 1b, 2, 3*	[60]
	*AtGRDP2*		*A. thaliana*	T	*-*	*-*	[61]
	*GRP7*	8	*A. thaliana*	S	*-*	*-*	[46]
	*-*	37-Ga 32-Gr	*G. arboreum* *G. raimondii*	-	*GaRB-GRP4, 17, 9,* *GrRB-GRP31,9*	*GrRB-GRP27,14*	[47]
	*AtZFP1*	-	*A. thaliana*	T	*AtZFP1 (At2g25900)*		[62]
	*BrRZ1,2,3*	-	*B. rapa*	S	*BrRZ1, BrRZ2, BrRZ3*		[21]
	*TaRZ1, 2,3*	-	*T. aestivum*	S	*-*		[49]
	*SRP1*	-	*A. thaliana*	S	*-*		[24]
	*CSDP1*	-	*A. thaliana*	S	*-*		[50]
	*CSDP2*	-	*A. thaliana*	T	*-*		[50]
	*CspA*	-	*E. coli/A. thaliana*	T	*-*		[51]
PPR	*wsl*	477	*O. sativa*	S (M)	responsive		[63]
	*PGN*	-	*A. thaliana*	S (M)	*-*	*-*	[64]
	*SOAR1*	-	*A. thaliana*	T	*-*	*-*	[52]
	*PPR40*		*A. thaliana*	T			[65]
	*-*	491	*O. sativa*		*LOC_Os05g47510, LOC_Os11g37330, LOC_Os03g53170*	*-*	[53]
	*PPR96*	105-E-type	*A. thaliana*	S (M)	*-*	*-*	[66]
	*-*	626	poplar		*PtrPPR5, 41,121, 185, 277, 481, 574*	*PtrPPR8, 30, 119*	[67]
	*-*	179 DYW-PPR	*Glycine max*	-	*GmPPR4*	*-*	[54]
DEAD-RH	*AtRH17*	-	*A. thaliana*	T	*-*	*AtRH9, AtRH25*	[68]
	*AtRH9, 25*	-	*A. thaliana*	S	*-*	*-*	[69]
	*OsRH58*	-	*A. thaliana*	T	*OsRH58*	*-*	[19]
	*SlDEAD31*	-	*S. lycopersicum*	T	*SlDEAD30, 31*	*-*	[56]
	*STRS1, STRS2*	-	*A. thaliana*	S	*-*	*-*	[70]
	*OsSUV3*		*O. sativa*	T			[71]
	*OsABP*		*O. sativa*	-	*OsABP*		[57]
KH	*hos5-1*		*A. thaliana*	S (M)	*-*	*-*	
PUF	*APUM5*		*A. thaliana*	S			[58]
	*SAHY9/APUM23*		*A. thaliana*	S			[72]
SDP	*At1g12800*	-	*A. thaliana*	S (M)	*-*	*-*	[73]
	*OsRBD1*	-	*O. sativa*	T	*-*	*-*	[74]
Ds-RBPs	*FRY2/CPL1*	-	*A. thaliana*	S (M)	*-*	*-*	[75]
	*DRB2,3*	-	*A. thaliana*	T	*-*	*-*	[76]
CRM	*CFM4)*	-	*A. thaliana*	S (M)	*-*	*-*	[77]
	*-*	14	*O. sativa*	-		*Os04g0464800, Os11g0592400,* *Os08g036010, Os05g0551900, Os09g0363100,* *Os06g0304500,* *Os01g0958400, Os04g0492900,* *Os05g0145500,* *Os08g0188000,* *Os08g0174900,* *Os01g0323300,* *Os01g0495900,* *Os10g0512500*	[78]
hnRNP	*-*	-	*E. guineensis*	-	*EgRBP42*	*-*	[25]
	At3g54770	-	*thaliana*	S	*-*	*-*	[28]
	*Tudor-SN*	-		S (M)	*-*	*-*	[79]

Note: T, stress tolerance; S, stress sensitive/susceptible; T (M), stress-tolerant mutant; S (M), stress-sensitive mutants; “-”, not available.

**Table 3:** Plant RNA binding proteins implicated in heat/high-temperature stress responses.

RBP Domain	Study	Total Genes	Species	Heat	Modulation		Ref.
					Upregulated	Downregulated	
YTH	*-*	39	*T. aestivum* L	-	responsive		[39]
	*-*	10	*C. sinensis*	-	*CitYTH2, 4, 5, 9*	*CitYTH3, 6, 7, 10*	[40]
	*-*	26	*M. domestica*	-	responsive		[42]
	*-*	13	*A. thaliana*	-	*AtYTH07, 10*	*AtYTH08*	[18]
	*-*	12	O. sativa	-	*OsYTH08*	*OsYTH11, 12*	[18]
SR	*-*	19	*S. lycopersicum*	-	*RSZ and RS2Z* *subfamilies,* *Sl-RS28, 29, 42, 46a*	*Sl-RS41*	[36]
	*-*	18	*B. distachyon*	-	responsive		[44]
	*-*	28	*B. rapa*	-	*BrSR3, BrRS2Z2, BrRSZ1, BrSCL2,3, 4, BrSR-like 3*	*BrRS2Z1, BrSCL5, BrRS1,2*	[20]
PUF	*APUM9*	*-*	*A. thaliana*	T	*-*	*-*	[80]
GR-RBP	*NtRGP-1a, 1b, 2,3*	*-*	*N. tabacum*	-	*NtRGP-1a,1b,2,3*	*-*	[60]
DEAD-RH	*OsRH58*	*-*	*A. thaliana*	-	*OsRH58*	*-*	[19]
	*SlDEAD31*	-	*S. lycopersicum*	-	*SlDEAD31*	*-*	[56]
	*STRS1, 2*	*-*	*A. thaliana*	S	*-*	*-*	[39]
SDP	*At1g12800*	*-*	*A. thaliana*	S	*-*	*-*	[73]
KH	*RCF3*	*-*	*A. thaliana*	T (M)	*-*	*-*	[81]
	*esr1-1, esr1-2*	*-*	*A. thaliana*	T (M)	*-*	*-*	[13]
hnRNP		-	*E. guineensis*	-	*EgRBP42*	*-*	[25]
	*FCA*	*-*	*A. thaliana*	T	*-*	*-*	[82]

Note: T, stress tolerance; S, stress sensitive/susceptible; T (M), stress-tolerant mutant; S (M), stress-sensitive mutants; “-”, not available.

**Table 4:** Plant RNA binding proteins implicated in cold/low-temperature stress responses.

RBPs Class	Study	Total Genes	Species	Cold	Modulation		Ref.
					Upregulated	Downregulated	
YTH	*-*	39	*T. aestivum* L	-	responsive		[39]
	*-*	10	*C. sinensis*	-	*CitYTH1, 2, 3, 4, 5, 6, 7, 8, 9, 10*	*-*	[40]
	*-*	5	*C. sativus*	-	*CsYTH1, 2, 4*	*-*	[38]
	*-*	26	*M. domestica*	-	*MdYTP1, 2, 5, 11, 14*	*MdYTP3, 4, 8, 15*	[42]
	*-*	13	*A. thaliana*	-	*AtYTH05*	*AtYTH10*	[18]
	*-*	12	*O. sativa*	-	*OsYTH08*	*OsYTH10, 5, 6, 7, 9*	[18]
SR	*-*	18	*B. distachyon*	-	responsive		[44]
	*-*	28	*B. rapa*	-	*BrRSZ3, BrSR1,2,4,* *BrRS2Z2-5, BrRSZ1,* *BrSCL4,5, BrSR-like 2*	*BrSR3,* *BrSCL2,* *BrSR-like 3*	[20]
GR-RBP	*NtRGP-1a, 1b,2, 3*	*-*	*N. tabacum*	-	*NtRGP-1a, NtRGP-1b, NtRGP-2, NtRGP-3*	*-*	[60]
	*HvGRRBP1*	*-*	*H. vulgare L.*	T	*HvGRRBP1*	*-*	[31]
	*OsGRP1, 4, 6*	*-*	*Oryza sativa*	T	*-*	*-*	[83]
	*atRZ-1a*	*-*	*A. thaliana*	T	*-*	*-*	[84]
	*OsRZ2*	*-*	*Oryza sativa*	T	*-*	*-*	
	*BrRZ1, Z2, Z3*	*-*	*B. rapa*	S	*BrRZ1, BrRZ2, BrRZ3*	*-*	[21]
	*atRZ-1a*	*-*	*A. thaliana*	T	*-*	*-*	[84]
	*TaRZ1*	*-*	*T. aestivum*	S	*-*	*-*	[49]
	*CP31A, CP29A*	*-*	*Arabidopsis*	T	*-*	*-*	[23]
	*OsCSP1, 2*	*-*	*O. sativa*	T	*OsCSP1, OsCSP2*	*-*	[85]
	*CSDP1*	*-*	*A. thaliana*	T	*-*	*-*	[9]
	*AtCSP2*	*-*	*A. thaliana*	S	*-*	*-*	[86]
	*AtCSP3*	*-*	*A. thaliana*	-	*AtCSP3*	*-*	[87]
PPR	*SOAR1*	*-*	*A. thaliana*	T	*-*	*-*	[52]
DEAD-RH	*RH50*	-	*A. thaliana*	S (M)	*-*	*-*	[88]
	*AtRH9, AtRH25*	-	*A. thaliana*	-	*AtRH9, AtRH25*	*-*	[68]
	*AtRH7/PRH75*	*-*	*A. thaliana*	T	*AtRH7/PRH75*	*-*	[89]
	*TCD33*	*-*	*O. sativa*	S (M)	*-*	*-*	[90]
KH	*sh1*		*A. thaliana*	S (M)	*-*	*-*	[91]
CRM	*CFM4*	*-*	*A. thaliana*	S (M)	*-*	*-*	[77]

Note: T, stress tolerance; S, stress sensitive/susceptible; T (M), stress-tolerant mutant; S (M), stress-sensitive mutants; “-”, not available.

## 3. Plant RBP Signatures/Domain Characteristics and Their Role in Abiotic Stress Responses

### 3.1. Glycine-Rich RBPs

Glycine-rich RBPs are class IV glycine-rich proteins (GRP) [48,92] and are characterized by the presence of the RNA recognition motif (RRM) or cold-shock domain (CSD) motifs at the N-terminus and a glycine-rich region at the C-terminus [4]. Based on domain characteristics, glycine-rich RBPs can be categorized into IVa (RRM motif), IVb (RRM and a CCHC zinc-finger motif), IVc (CSD and two or more zinc-finger motifs), and IVd (two RRMs) subgroups [47]. In general, GR-RBPs are functionally conserved across plant species. For example, the cold-sensitive phenotypes of *atgrp7* were successfully rescued by *OsGRP1* and *OsGRP4* of rice [83], while another gene, *OsGRP6,* conferred freezing tolerance in the *atgrp7* plants. *AtGRDP2* overexpression improved salt stress tolerance in Arabidopsis and improved growth by increasing the indole-3-acetic acid levels in transgenic lines [61]. Contrastingly, overexpression of *AtGRP7* exhibited salinity- and drought-sensitive phenotypes in Arabidopsis while improving freezing tolerance [46]. atRZ-1a act as RNA chaperones during cold stress and contribute to cold tolerance in Arabidopsis [93]. An attempt to identify the GR-RBPs in two important cotton species, *Gossypium arboreum* and *Gossypium raimondii,* unearthed 37 *GaRB-GRP* and 32 *GrRB-GRP* genes, respectively [47]. At least eight of those *GaRB-GRP* and *GrRB-GRP* genes showed differential expression under salt stress conditions indicating they might be involved in salt stress responses of cotton species. The *HvGR-RBP1* of barley was upregulated in response to cold stress [31]. A recent study investigating the functions of *OsGRP3* in rice using overexpression and knockout mutants revealed that *OsGRP3* expression contributes to drought tolerance by alleviating the ROS accumulation through the regulation of ROS genes. In contrast, mutants exhibited vulnerability to drought stress conditions [48]. The functions of two important subclasses of glycine-rich RBPs are separately presented below.

#### 3.1.1. Zinc Finger Containing Glycine-Rich RBPs/Zinc Finger RBPs

Zinc finger RBPs belong to IVb subgroups of glycine-rich RBPs and have a CCHC-type zinc finger between the N-terminal RRM and the glycine-rich C-terminal [21] designated as RZ (Xu et al., 2014). The genome-wide analysis identified 103 CCCH genes in *B. rapa* [94]. Of these, 12 of 17 RR-TZF genes showed that they were remarkably upregulated by salt or mannitol-induced drought stress conditions suggesting their potential role in stress responses. BraA10g029760 and BraA05g005940 were easily induced by salt stress, while BraA09g020370 was strongly induced by drought stress (Table 1 and Table 2). The nuclear-localized *BrRZ1*, *BrRZ2,* and *BrRZ3* of Chinese cabbage were significantly induced by multiple abiotic stresses like cold, drought, and high salinity stress conditions [21]. However, transgenic overexpression of *BrRZ2* and *BrRZ3* in Arabidopsis generated sensitive phenotypes under salt, drought, and cold stresses. Characterization of the wheat zinc finger-containing glycine-rich RNA-binding proteins (RZs) *TaRZ1*, *TaRZ2*, and *TaRZ3* using transgenic overexpression Arabidopsis lines provided differential sensitivity to abiotic stress conditions [49]. All three (*TaRZ1*, *TaRZ2*, and *TaRZ3*) lines showed germination susceptibility under salt stress conditions compared to wild-type lines. Similarly, TaRZ2 or TaRZ3 lines were sensitive to drought. Interestingly, the expression of TaRZ2 conferred freeze tolerance in Arabidopsis. AtZFP1 was shown to be positively associated with salt resistance in Arabidopsis [62].

#### 3.1.2. Cold Shock Domain Protein (CSDP)

Unlike bacterial CSDPs, typical plant CSDPs have a glycine-rich region interspersed with a CCHC-type zinc finger motif at the C-terminal, in addition to the characteristic N-terminal cold shock domain (CSD) [86,95]. The bacterial CSD domain comprises 70 AA residues, and it can bind to RNA targets through its inherent RNA binding motifs (RNP-1 and RNP-2) [86]. Overexpression of *AtCSP2* significantly decreased freezing tolerance when cold-acclimated, while *atcsp2* mutant improved freezing tolerance through upregulation of CBF transcription factors and downstream genes in the cold stress pathway [86]. In another study, overexpression of *CSDP1* in Arabidopsis decreased the cold and drought tolerance, while *CSDP2* enhanced the salt tolerance in germinating seedlings [50]. Moreover, both *CSDP1* and *CSDP2* complement the cold-sensitive phenotypes in *atgrp7* mutant seedlings suggesting their positive association with cold stress tolerance. In another study, overexpression of *AtCSP3* in transgenic plants contributed to cold acclimation processes [87].

Similarly, the cold adaptation properties of *OsCSP1* and *OsCSP2* of rice were tested with cold-sensitive bacterial strains and it was found that both genes can compensate for the loss of bacterial CSP genes, suggesting they are essential for cold stress adaptation mechanisms in plants [85]. Attempts were also made to utilize the bacterial cold-shock domain proteins for improving tolerance to abiotic stresses in plants. One such example is the overexpression of codon-optimized *CspA* and *CspB* (from *E. coli*) in Arabidopsis, which yielded drought and salt stress tolerance [51].

Interestingly, several stress-response genes were upregulated in those transgenic lines, which positively corresponded with stress tolerance characteristics under stress conditions. A natural variation in the coding region of *BoCSDP5, Brassica oleracea* cold shock domain protein 5 was found to be associated with the low-temperature stress tolerance of cabbage [96].

### 3.2. Serine/Arginine-Rich (SR) Domain

SR proteins are non-small nuclear ribonucleoproteins (non-snRNP) which form complex spliceosome machinery that mediates numerous events in pre-mRNA splicing [20,97]. The canonical SR domain proteins consist of one or two N-terminal RNA recognition motifs (RRMs), which bind to the RNA targets, and a serine-arginine or arginine-serine dipeptide-rich C-terminal-terminal region (RS domain) with a variable length [36,43,97]. Based on the domain organization structures and the presence of amino acid sequence motifs, SR proteins can be classified into six subfamilies: SR (two RRM), RS (two RRM, but the second RRM lacks SWQDLKD motif), SC (with just 1 RRM), SCL (SC-like with charged N terminal AA residues), RSZ (CCHC zinc knuckle), and RS2Z (two CCHC zinc knuckles) [44,98,99]. Of these, three subfamilies (SR, SC, and RSZ) were found only in plants. Additionally, a few SR-like genes (e.g., SR45, SR45a) were found to have two RS domains instead of the one found in typical SR proteins [45,100,101]. Genome-wide identification of SR proteins in *Brassica rapa* [20], wheat, *Brachypodium distachyon* [44], tomato [36], and Cassava [43] resulted in 28, 40, 18, 19, and 18 SR genes, respectively. Of these, the expression of 22 and alternative splicing patterns of 17 genes in *B. rapa* were significantly altered in response to cold, heat, or oxidative stresses [20]. Similarly, 21 genes of wheat were differentially regulated by drought, cold, heat, and salt stress conditions [44]. Heat stress (37.5 °C) enhanced the expression of six tomato SR genes (*Sl-RS28*, *Sl-SR32*, *Sl-SR33*, *Sl-SC30b*, *Sl-RS2Z36*, and *Sl-SR46a*) in leaves (Table 3). Overexpression of the cassava SR gene *MeSR34* in Arabidopsis helped plants maintain reactive oxygen species homeostasis and increased the expression of osmotic stress-related genes, which eventually led to salt tolerance [43].

In addition to post-transcriptional regulations, SR proteins are also involved in protein modifications. For example, *SF2/ASF* was shown to induce protein sumoylation in response to heat shock stress [102]. Salt stress induces alternative splicing of *SR45a* in Arabidopsis and produces two variants, designated as SR45a-1a and SR45a-1b. Transgenic lines with overexpression of both variants of SR45a exhibited susceptibility to salt stress [34]. Further, it was shown that *SR45a* is essentially important for alternative splicing and mRNA maturation of several salt-tolerance genes. In *B. rapa*, *BrSR45a* was induced by drought stress, and the overexpression of *BrSR45a* improved drought tolerance in Arabidopsis [45]. Further analysis revealed that *BrSR45a* overexpression resulted in alternative splicing of drought-stress response genes and *BrSR45a* interacting genes. A recent study [59] showed that AtSR45.1, one of the two alternative splice variants produced by *AtSR45*, can complement the salt-sensitive phenotypes of *atsr45* mutants but is not rescued by AtSR45.2. These results indicate the biological significance of splice variants and the role of SR proteins in stress-responsive alternative splicing. Considering SR proteins importance in plant stress responses, a recent study generated 22 rice SR gene mutants/knockout lines for precise understanding of their biological significance [103]. The functional characterization of those mutants will be expected to add more to our understanding of rice SR functions in the near future.

### 3.3. PPR Proteins

Pentatricopeptide-repeat proteins (PPR) are nuclear-encoded proteins constituting a large gene family in higher plants. They are characterized by tandem arrays of degenerate 35-amino-acid (PPR motifs) [53]. The canonical PPR motif (a hairpin of two α-helices) can recognize single-stranded RNA targets. This α-solenoid RNA-binding proteins (RBPs) superfamily can be classified into P (P motifs only) and PLS (P, P motif variants (L, Long; S, Small motifs), E or DYW (non-P motif)) subfamilies. However, the PPR classification has been revisited recently, which expanded the subgroups of P and PLS PPR subfamilies based on the existence of C-terminal domains. Detailed information on PPR subgrouping can be found elsewhere [53,104]. Of these, P-type PPR subfamilies were shown to function in RNA splicing, stabilization, and translational activation, while PLS-type PPRs are primarily involved in RNA editing [105]. In general, PPR proteins were critical for post-transcriptional regulation of chloroplast and mitochondrial gene expression [64,105]. For instance, a chloroplast-localized PPR mutant, white stripe leaf (*wsl*) in rice, appeared with a splicing defect in plastid transcripts and reduced gene protein expression (e.g., PEP-dependent plastid gene expression) [63]. The *wsl* phenotyping analyses revealed that *wsl* is hypersensitive to salinity and ABA, while loss of function of mitochondria-localized pentatricopeptide repeat protein for germination on NaCl (PGN) increases the accumulation of reactive oxygen species and endogenous ABA levels, which possibly leads to their susceptibility to salt stress [64]. Interestingly, ectopic expression of PGN did not alter the salt-sensitive phenotypes, suggesting that PPR proteins are tightly regulated in plant organelles. On the contrary, overexpression of cytosol-nucleus dual-localized PPR protein, *SOAR1,* strongly promoted drought, salt, and cold tolerance during seed germination and postgermination growth periods in Arabidopsis [52]. Genome-wide analysis in rice, poplar, and soybean identified a total of 491 (subfamily, 246-P;245 PLS), 626, and 179 (DYW subgroup) PPR genes, respectively [53,54,67]. The large presence indicates that PPR functions are essential for normal plant growth and stress responses. Expression profiling of many PPR genes in rice was found to be differentially expressed under drought or salinity stress [53]. Many P-type PPR genes were altered in poplar plants in response to cold (122 PPRs) and salt [67]. In an attempt to improve drought tolerance, *GmPPR4* was overexpressed in soybean. The *GmPPR4* transgenic plants confer drought tolerance, delayed drought symptoms, reduced oxygen radicals, and increased proline content [54]. Additionally, several drought inducible genes were relatively overexpressed in transgenic lines. Arabidopsis is said to have 105 E subgroup PPR genes. Of these, loss of function of *PPR96* leads to salt-hypersensitive phenotypes [66], suggesting its importance in salt stress responses. While *PPR40* overexpression promotes salt tolerance [65], inactivation of *PPR2 (ppr2)* in Arabidopsis is responsible for heat-sensitive phenotypes [106]. Under cold stress, *TCD10* is essential for chloroplast development in rice [16]. Similarly, drought sensitivity was found to be increased in PRECOCIOUS1 (POCO1) Arabidopsis mutant (*poco1*) plants [55]. The mitochondrial PRECOCIOUS1 (POCO1) is generally involved in ABA signaling and flowering time regulation. Apart from organelle-specific functions, PPR was also found to have developmental stage-specific functions. For example, mitochondrial RNA editing factor *slo2* mutants increased salt susceptibility during germinations; however, *slo2* increased salt and drought stress tolerance in adult plants [107].

### 3.4. YTH Domain

The YTH (YT521-B homology) domain-containing RBPs comprised of 100–150 highly conserved amino acids at the C-terminal that are rich in aromatic residues, can bind to m^6^A-containing mRNAs [18,38], and regulate the processing of mRNAs for their destined function in plants according to the plant requirements. Genome-wide analysis of YTH domain protein in common wheat, *Citrus Sinensis*, *Cucumis sativus*, apple, Arabidopsis, and rice identified 39, 10, 5, 22–26, 13, and 12 genes, respectively (Table 1). In a first attempt, the overexpression of the apple YTH domain-containing genes, MhYTP1 or MhYTP2 in Arabidopsis, enhanced salinity and drought tolerance [108]. Additionally, both of these were shown to participate in low- and high-temperature stresses [109]. Consistent with previous results, *MhYTP1* overexpression in apples promoted drought tolerance through elevated ABA content, increased stomatal density, reduced stomatal aperture, enhanced the net photosynthesis rate, increased biomass, and increased water use efficiency (WUE) under drought conditions [41]. The cis-regulatory elements of YTH domains and abiotic stress gene expression analyses revealed that YTH proteins might participate in heat, drought, salinity, and cold stresses [18,38,39,40,41,42]. One of the isoforms of the polyadenylation factor was designated as CPSF30-L, which has YTH domain, and was shown to influence the expression of multiple abiotic stress genes in Arabidopsis [110]. Being relatively new classes of RBPs, future studies focus on their role in abiotic stress responses expected to bring more information about the biological significance of YTH domain proteins in plants. Identifying target m^6^A mRNAs and their potential interaction with YTH domains will also provide a potential opportunity to manipulate them for better agronomic traits in plants.

### 3.5. Pumilio/Fem-3 Binding Factors (PUF) RBPs

Pumilio proteins lack typical RRM or K-homology domains. The characteristic features of this class of RPBs are tandem repeats of 8 PUF/Pumilio homology domains [111]. Each Puf domain contains 35–39 amino acids and recognizes one RNA base [112]. Canonical PUF proteins can bind to cis-regulatory elements (conserved UGURN_1–3_AU (A/U) motif in the 3′UTR) of mRNA targets to modulate their regulation post-transcriptionally [8,10]. PUF proteins also function as translational repressors and are highly conserved among plants [113]. *APUM5* overexpression in Arabidopsis resulted in salt- and drought-sensitive phenotypes [58]. Subsequent analyses of *APUM5* transgenic lines revealed that the abiotic stress-responsive genes were negatively regulated, confirming the negative regulatory functions of *APUM5* at transcriptional levels. In another study, the loss of function of *APUM23* mutant had salt-sensitive phenotypes. The detailed analysis of biochemical and molecular changes in those phenotypes showed that ABA contents were lowered [72]. In addition, the expression of ABA-associated genes like NCED3, ABI2, PP2CA, and major ABA-responsive marker genes, such as RD20 and RD29B, were found to be down-regulated. However, the exogenous application of ABA complemented a hypersensitive response to high salinity stress indicating that *APUM23* participates in ABA biosynthesis and abiotic stress-responsive gene expression in Arabidopsis. Functional and molecular characterization of *APUM9* further confirms the negative gene regulation of PUF domain-containing proteins, just like their animal and yeast counterparts [80]. APUM9 binding with DCP2, a catalytic subunit of decapping complex, was shown to be responsible for the rapid decay of DCP2. Interestingly, overexpression of *APUM9* produced relatively better heat stress-tolerant phenotypes.

### 3.6. DEAD-Box RNA Helicases

The DEAD-box RNA helicases (DEAD-box RHs) are members of the SF2 RNA Helicase family and represent the largest family of RNA helicases [56]. The characteristic helicase domain consists of at least nine conserved motifs, including a Walker B motif/Motif II with characteristic DEAD (Asp–Glu–Ala–Asp) sequences [15,70,89]. DEAD-box RNA helicases are ATP-dependent RNA binding proteins and RNA-dependent ATPases [114]. DEAD-box RHs are known to participate in RNA metabolism, ribosome biogenesis, and translation [88]. The Arabidopsis transgenic lines that overexpressed *AtRH17* displayed tolerance to NaCl consistently across different developmental/growth stages [68]. Interestingly, analysis of transcriptomic dynamics of ABA-dependent and ABA-independent pathways in transgenic lines revealed no changes, thus revealing the existence of unidentified other pathways. An earlier study with *atrh7* mutant demonstrated cold hypersensitivity phenotypes and decreased expression of potential cold-tolerance candidate genes [89], suggesting that *AtRH7* may be crucial for cold tolerance. A study in tomato plants identified two stress-response genes, designated as *SlDEAD30* and *SlDEAD31*. The expression of both genes was induced in salt stress conditions [56], while the expression of *SlDEAD31* was also enhanced by other stresses, including drought/dehydration, cold, and heat. The phenotyping of *SlDEAD31* overexpression transgenic lines dramatically enhanced salt tolerance and slightly improved drought resistance by modulating the expressions of multiple biotic and abiotic stress response genes. A chloroplast-localized rice DEAD-RH gene, designated as *OsRH58,* was induced at transcript levels by multiple abiotic stress conditions like drought, salt, and heat, decreased by cold stress conditions [32].

Further analysis with the *OsRH58*-overexpressing Arabidopsis plants displayed improved salt and drought tolerance by increasing the translation of plastid mRNAs. The Arabidopsis *rh50* mutant reduced cold tolerance efficiencies, indicating its importance in regulating cold stress responses [88]. In some cases, DEAD-RH proteins underwent stress-induced relocalization in plant cells. A study by Asif Khan et al. [70] found that two genes of Arabidopsis, known as stress response suppressor1 (STRS1) and STRS2 exhibited relocalization in response to various stresses and abscisic acid (ABA). Transgenic overexpression of STRS reduced stress tolerance, indicating their roles as attenuators of abiotic stress responses. Fittingly, *strs1* and *strs2* mutants displayed tolerance to abiotic stresses. In response to salt or drought stress, *AtRH9* and *AtRH25* were downregulated [69]. The transgenic Arabidopsis overexpressing *AtRH9* and *AtRH25* revealed poor germination under salt stress conditions, suggesting the possibility of *AtRH9* and *AtRH25* being the negative regulators of salt stress tolerance. Salt and drought stress upregulates the expression of *OsABP* in rice [57]. Overexpression of *OsSUV3* is shown to improve salinity stress tolerance [115]. A recent study investigating the thermo-sensitive chlorophyll-deficient rice mutant *tcd33* demonstrated that functional TCD33 is crucial for early chloroplast development, regulation of cold-responsive genes, and cold tolerance in rice [90].

### 3.7. KH Domain

The heterogeneous nuclear ribonucleoprotein K (hnRNP K) homology (KH) domain protein is the second most frequently found RNA-binding domain after RRM. Multiple copies (up to 15) of KH Domains can be found in a protein, and each KH domain has a highly conserved consensus sequence (VIGXXGXXI) in the middle of a 60 AA long chain with a typical pattern of hydrophobic residues [116]. A KH domain-containing protein can bind RNA or single-stranded DNA to regulate transcriptional and post-transcriptional gene regulations [13]. The Arabidopsis KH-Domain RNA-Binding Protein *ESR1* insertional knockout mutants *esr1-1* and *esr1-2* confer increased heat tolerance by altering the expression of several abiotic and biotic stimuli genes [13].

The *hos5-1* mutants were shown to have sensitive phenotypes against salt stress and ABA [117]. Under salt treatments, the color of the leaves of *hos5-1* was more yellowish than that of the wild-type seedlings. Another KH domain protein named SHINY1 (*SH1*) was evaluated for its response to cold stress. The phenotyping results showed that *sh1* mutants are more susceptible to cold stress during vegetative growth [91]. RCF3 is a negative regulator of most HSFs, including HSFA1a, HSFA1b, and HSFA1d. Consistent with that, the loss of function of *rcf3* mutants conferred improved heat stress tolerance [81].

### 3.8. S1 Domain Containing-Protein (SDP)

S1 domain-containing proteins (SDPs) harbor the S1 RNA binding domain as their RNA binding module that controls chloroplast gene expression [73]. The S1 RNA binding domain (~70 AA), which was originally identified in the ribosomal protein S1 (RPS1) of *E. coli,* had six copies of S1 domain repeats, and the numbers of S1 domain repeats in SDPs of different species are not similar [15]. In plants, SDP proteins are targeted to chloroplast and involve in chloroplast ribosomal RNA processing through binding with ribosomal RNAs 23S, 16S, 5S, and 4.5S [17]. The defective SDP (At1g12800) of *sdp* Arabidopsis mutant lines attributed to increased sensitivity to salt, heat, and freezing stress [73]. Further analysis showed impaired chloroplast translation in mutants, affecting the expression of stress response nuclear genes against abiotic stresses. A plastid ribosomal protein S5 (*RPS5*) overexpression exhibited cold tolerance in Arabidopsis [118]. Consistently, the *rps5* mutant produced cold-sensitive phenotypes by decreasing the expression of several cold-responsive proteins with others.

### 3.9. The Chloroplast RNA Splicing and Ribosome Maturation (CRM) Domain-Containing Proteins

The chloroplast RNA splicing and ribosome maturation (CRM) domain-containing proteins can carry multiple copies of CRM domains [77]. The conserved GxxG sequences in the loop of CRM domains are responsible for the RNA binding ability of CRM domain-containing proteins [15]. Based on the number of CRM domains and their structure, CRM domain-containing proteins can be classified into the CRS1 subfamily, CAF subfamily, subfamily 3, and subfamily 4 [78]. A study involving the characterization of CRM domain protein, designated CFM4, in *Arabidopsis thaliana* revealed that loss of function of CFM4 hampered the stress tolerance efficiencies against drought, salt, and cold conditions [77]. The functional characterization of the other CRM domain protein of Arabidopsis (mitochondrial-targeted, *CRM9*) revealed that the loss of function of *CRM9* increased the defective-growth phenotypes in the *crm9* mutant under high salinity and drought conditions. Restoring the functions of *CRM9* helped the plants to gain their normal phenotypes [119]. In rice, 14 CRM domain proteins, which are abundantly expressed in leaf tissues, were transcriptionally downregulated by salt stress [78]. No such clear expression pattern was observed for drought and cold stress conditions.

### 3.10. Double-Stranded RNA-Binding Protein (DRBP)

This class of RBPs harbors an evolutionarily conserved, double-stranded RNA (dsRNA)-binding domain (dsRBD)/dsRNA-binding motif (dsRBM) [76]. Plant proteins can have one or more DSRM, and each is comprised of 65 conserved amino acids with a characteristic (α1-β1-β2-β3-α2) fold that interact with double-stranded RNAs [12,76]. Studies with Arabidopsis DRBPs revealed their association with small plant RNA biogenesis pathways [120]. The loss of function of FIERY2 (FRY2), which harbors two double-stranded RNA binding domains in *fry2* mutants, exhibited salt tolerance during germination [75] while being more susceptible to freezing stress. Five DRBPs (DRBP1-5) were reported in Arabidopsis. Of these, overexpression of *DRB2* and *DRB3* exhibited salt tolerance [76]. Upon exposure to cold stress conditions, *drb2* and *drb3* mutants enhanced the accumulation of anthocyanins by regulating anthocyanin biosynthesis genes. Another DRBP, designated as hyponastic leaves1 (*HYL1*), was characterized for its role in endoplasmic reticulum (ER) stress, which is a result of the accumulation of misfolded/unfolded proteins in ER [121]. The loss of function of *HYL1* in the *hyl1* mutant was shown to be more sensitive to tunicamycin, a causative agent of ER stress. Previously it was demonstrated that *hyl1* is hypersensitive to the stress hormone abscisic acid (ABA) [122], suggesting its possibility in ABA-dependent abiotic stress response pathways.

## 4. Conclusions and Future Directions

We presented a comprehensive overview of plant RBP proteins and their conservative RBDs that are useful in RBP–RNA interactions. We also presented compelling evidence that both canonical and non-canonical RBPs play a role as transcriptional regulators in the drought, heat, high salinity, and cold stress responses of plants. Additionally, several RBP genes modulated by abiotic stresses were collected as resources that may provide the impetus to conduct more studies concerning their biological significance in stresses. Among the latest tools, the application of RIC for the discovery of RBPs amassed several hundreds of canonical and novel candidate RBPs across plant genomes. Although recent advances in genome biology have revolutionized our notion of plant RBPs and experiments concerning the discovery of RBPs are steadily increasing, a lot remains to be uncovered: the target specificity of RBPs, motif structures that constitute the novel/non-canonical RBDs, the primary factors that control the stress response RBPs, and the functional roles of individual RBPs all need to be elucidated. While the census of plant RBPs is rapidly expanding, experiments dealing with the functional characterization of RBPs are not expanding equally due to time-consuming practical approaches and technical shortcomings. The application needed is a relatively simple technique, yet precise gene editing/knockout tools like CRISPR/Cas are meagerly utilized in the functional characterization of plant RBPs. In addition, the RIC has been preferentially used in model plants. It needs to be expanded to several other model and non-model plants in the presence of abiotic stress conditions that hamper the key agronomic traits. Adding the structural data of novel RBPs is inevitable in understanding their interaction with target mRNAs and their target specificity. The genetic manipulation techniques for agronomic trait improvement would use RBPs to target a chosen RNA sequence. Considering the increasingly worsening climate change and its adverse effects on crops, future work focusing on new insights into stress-responsive RBPs will benefit the plant biotechnologist in developing climate-resilient crops.

## Data Availability

Not applicable.
